# Recurrent primary haemangiopericytoma of the bladder: A case report and literature review

**DOI:** 10.3892/ol.2014.1862

**Published:** 2014-02-07

**Authors:** LIWEI XU, GUOQING DING

**Affiliations:** Department of Urology, Sir Run Run Shaw Hospital, College of Medical Sciences, Zhejiang University, Hangzhou, Zhejiang 310016, P.R. China

**Keywords:** haemangiopericytoma, bladder, recurrent, primary

## Abstract

Haemangiopericytoma (HPC) is a rare soft-tissue tumour with great histological variability and unpredictable clinical and biological behaviour. HPC of the bladder is exceedingly rare and carries uncertain malignant potential. The current study reports a case of HPC of the bladder in a 48-year-old female, who was admitted to the Sir Run Run Shaw Hospital (Hangzhou, China) due to a large bladder mass. The patient exhibited no mass-related symptoms, such as pain, gross haematuria or urinary irritation. Seven years prior to admission, the patient underwent surgical resection of a bladder mass, which was determined to be HPC. Computed tomography scans showed a well-defined, heterogeneously enhancing solid cystic mass in the bladder. The patient underwent complete excision of the tumour and a partial cystectomy. The histopathological diagnosis was HPC of the bladder. The post-operative recovery was uneventful and no evidence of recurrence or metastasis was identified during two years of follow-up. The clinical and histological features, and the treatment and prognosis of this tumour are discussed together in the present study, with a review of the literature.

## Introduction

The majority of primary bladder tumours are transitional cell (urothelial) tumours. Cases of squamous cell carcinoma, primary adenocarcinoma or small cell carcinoma are encountered much less frequently (1). Haemangiopericytoma (HPC) of the bladder, which is a tumour originating from the vascular pericytes of Zimmermann, is exceedingly rare and carries uncertain malignant potential. HPC mostly arises in the lower extremities, retroperitoneum and head and neck area (2–4). To the best of our knowledge, only eight cases of HPC of the bladder have been previously reported in the English literature (2–9). The rarity of HPC of the bladder makes it difficult to predict the clinical behaviour and determine the appropriate treatment of this tumour. The current study reports a case of recurrent primary HPC of the bladder. The clinical and histological features, and the treatment and prognosis of this tumour are discussed together with a review of the literature.

## Case report

**Figure 1:**
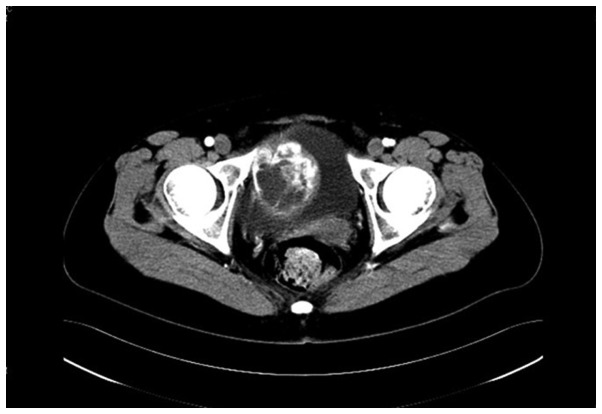
Computed tomography (CT) scan showing a well-defined heterogeneously enhancing 6.2×5.0-cm solid cystic mass in the bladder.

A 48-year-old female patient was admitted to the Sir Run Run Shaw Hospital (Hangzhou, China) due to a large mass in the bladder identified during a health examination in June 2011. The patient exhibited no mass-related symptoms, such as pain, gross haematuria or urinary irritation. Seven years prior to admission, the patient underwent surgical resection of a bladder mass, which was diagnosed as HPC of the bladder. However, the patient did not attend regular follow-up examinations after the surgery. The patient was otherwise healthy. Computed tomography (CT) scans showed a well-defined, heterogeneously enhancing 6.2×5.0-cm solid cystic mass in the bladder (Fig. 1). Cystoscopy showed extrinsic compression of the right bladder wall, but the bladder mucosa was normal. The physical examination was unremarkable. No lymph node or distant metastasis was found. Following a thorough pre-operative evaluation, the patient underwent complete excision of the tumour and a partial cystectomy. The tumour was 6.5×5.0×4.0 cm in size, with an intact external surface. The cut surface of the tumour was grey-white, with a medium texture.

**Figure 2:**
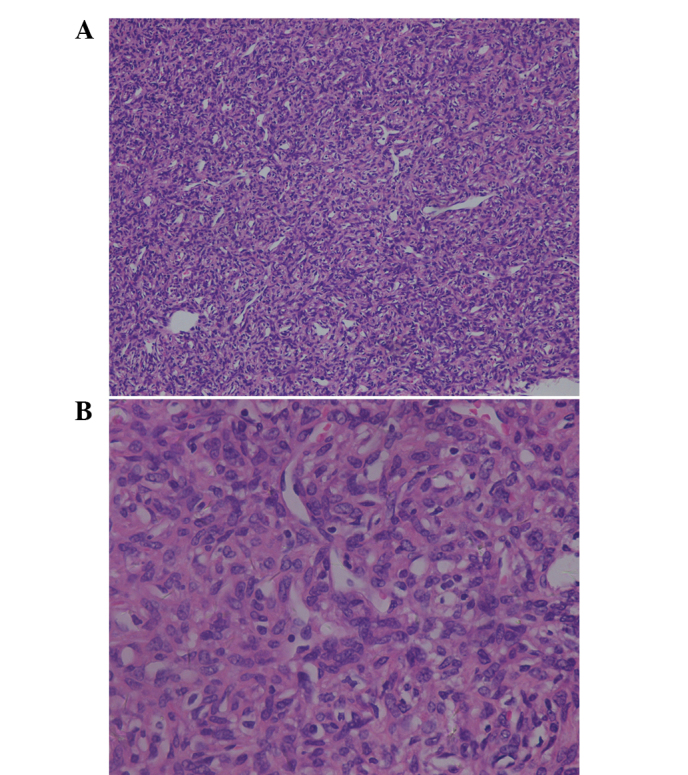
Histopathological examination revealed a neoplasm consisting of spindle-shaped cells, which were arranged around the vasculature, with a ‘staghorn’ configuration. Magnifications of (A) ×100 and (B) ×400 (haematoxylin and eosin staining).

The histopathological examination revealed a neoplasm consisting of spindle-shaped cells, which were arranged around the vasculature, with a ‘staghorn’ configuration (Fig. 2). The images captured were hypercellular and showed cells that exhibited oval nuclei. Mitosis was rare and necrosis was not present. The neoplastic cells exhibited marked positivity for Bcl-2 and were negative for cluster of differentiation (CD)34, CD31, factor VIII, epithelial membrane antigen, S100, chromogranin A, cytokeratin (CK)7 and CK19. The proliferation marker, Ki-67, was positive in <5% of the tumour cells. The histopathological diagnosis was HPC of the bladder and surgical margins were noted to be tumour-negative.

The post-operative recovery was uneventful, but the patient refused further adjuvant radiotherapy. To date, the patient has been followed up regularly with no evidence of recurrence or metastasis. Written informed consent was obtained from the family of the patient.

## Discussion

HPC is a rare soft-tissue tumour, first described by Stout and Murray in 1942 (10). HPC was considered to originate from the pericytes, a specific cell type that surround the capillary vessels. However, according to the World Health Organization for the classification of tumours of soft tissues and bone, the term ‘haemangiopericytoma’ may be used to refer to a variety of tumours, which have the presence of a thin-walled branching ‘staghorn’ vascular pattern and resemble cellular areas of solitary fibrous tumours (11). An accurate histopathological assessment determines the definitive diagnosis of HPC. There is not always clarity in the prediction of the clinical behaviour of HPC and this does not always correlate with the histopathological features of the tumour either. There is variation between studies with regard to the histopathological criteria for malignancy, and strict universal criteria have not yet been identified (12).

HPC in the urinary bladder is extremely rare. To the best of our knowledge, only eight cases of HPC of the bladder have been previously reported in the English literature (Table I) (2–9). In these cases and including the current study, the mean age of the patients at the time of diagnosis is 48.5 years (range, 29–72 years), with a predominance of females (six vs. two). The mean size of the tumours is 8 cm (range, 2.5–12 cm) and the clinical features of the patients are not characteristic. Urinary symptoms, such as haematuria and frequency, were noted in four patients and three patients had pain associated with the masses. Hypoglycaemia*,* attributed to the extensive metabolism of glucose within the tumour, was present in one case (6). In one patient, anaemia and weight loss were the reasons for hospitalisation (5). However, the patient of the current study had no complaints associated with the tumour.

Imaging is important in the diagnosis and management of HPC by demonstrating the vascular nature of the tumour and revealing the exact source of its blood supply, its size and its association with the adjacent organs. However, no characteristic signs of HPC have been recognised on ultrasonography, CT scan or magnetic resonance imaging. Commonly, previous studies have depicted a large mass, but with no pathognomonic features. Cystoscopy may reveal no intravesical pathology, but evidence of compression of the bladder wall.

The clinical and biological behaviour of HPC is variable and unpredictable. En bloc resection remains the cornerstone of therapy for curative intent (13). The surgeon must be as radical as possible to avoid incomplete tumour resection and a high frequency of relapse. Open surgery was used in seven of the patients in the previous studies, with the exception of one case, reported by Sutton *et al*, in which the patient underwent a transurethral resection and the tumour recurred two years later (4). Since HPC originates in the bladder wall, we do not recommend the transurethral approach for HPC of the bladder due to the fear of incomplete resection. An appropriate first surgical treatment must be selected to obtain a complete view of the mass. For tumours exhibiting evident criteria for malignancy, adjuvant radiotherapy should be considered (13). Radiotherapy is reserved as the adjuvant therapy in cases of incompletely excised lesions and recurrent and inoperable tumours. Radiotherapy was used in two of the previously reported cases and the authors considered radiotherapy effective for preventing recurrence and controlling the hypoglycaemic syndrome (2,6). Systemic chemotherapy may be employed for metastasis and recurrence. However, standard and effective chemotherapeutic regimens have yet to be established (14). Previously, one patient with HPC of the bladder received chemotherapy for metastases, but did not benefit from the treatment (7). In other HPCs in various locations, chemotherapy does not appear to be an effective adjunct therapy (15,16).

The outcomes of the previously reported cases were quite different: One patient succumbed three days after surgery from a pulmonary embolism caused by tumour thrombi; lung metastasis was noted in a patient nine years after surgery; and three patients developed local recurrence following the initial surgery (after seven years in two cases). Since recurrence and metastasis may occur after a number of years, lifelong regular follow-up is necessary. To date, the current patient has been followed for two years and no evidence of local recurrence or metastasis has been identified.

HPC of the bladder is an extremely rare tumour with unpredictable clinical and biological behaviour. Radical surgical excision is considered to be the cornerstone of treatment. Radiotherapy is reserved as the adjuvant therapy in cases of incompletely excised lesions and for recurrent or inoperable tumours. The efficacy of classical chemotherapy appears disappointing. In addition, since recurrence and metastasis may occur after a number of years, lifelong regular follow-up is necessary.

## Figures and Tables

**Table I:** Characteristics of patients with HPC of the bladder previously reported in the English literature, together with the current case (n=9).

First author/s (year)	Age, years	Gender	Size, cm	Symptoms	Treatment	Follow-up
Sezhian *et al* (2007)	52	F	12	Anaemia and weight loss	Total cystectomy and an ileal conduit	Well at two years
Kibar *et al* (2006)	45	M	4	Left groin pain, vague suprapubic discomfort and urinary frequency	Partial cystectomy and adjuvant radiotherapy	Well at two years
Soran *et al* (2007)	72	F	~12	Symptoms of hypoglycaemia	Local palliative radiotherapy	Succumbed at three years
Bagchi *et al* (1993)	NA	NA	NA	NA	NA	NA
Burgess *et al* (1993)	29	F	10	Right lower abdominal pain	Excision of the lesion	Well at six months
Sutton *et al* (1989)	30	F	6	Acute urinary retention	Complete transurethral resection	Recurrence at two years
Prout and Davis (1977)	40	M	12	Right groin pain, urinary frequency, dysuria and a right lower quadrant mass	Excision of the lesion and chemotherapy for metastases	Metastases at nine years
Baumgartner *et al* (1976)	72	F	2.5	Intermittent painless total haematuria with urinary frequency and vague suprapubic discomfort	Partial cystectomy with ligation of the ureter, hysterectomy and bilateral salpingo-oophorectomy	Succumbed at three days from a pulmonary embolism caused by tumour thrombi
Current case	48	F	6.5	No symptoms	Partial cystectomy	Well at two years

HPC, haemangiopericytoma; F, female; M, male; NA, not available.
